# Expression of lncRNAs in Low-Grade Gliomas and Glioblastoma Multiforme: An In Silico Analysis

**DOI:** 10.1371/journal.pmed.1002192

**Published:** 2016-12-06

**Authors:** Brian J. Reon, Jordan Anaya, Ying Zhang, James Mandell, Benjamin Purow, Roger Abounader, Anindya Dutta

**Affiliations:** 1 Department of Pathology, School of Medicine, University of Virginia, Charlottesville, Virginia, United States of America; 2 Department of Biochemistry, University of Virginia, Charlottesville, Virginia, United States of America; 3 Division of Neuro-Oncology, Neurology Department, University of Virginia Health System, Old Medical School, Charlottesville, Virginia, United States of America; Washington University School of Medicine, UNITED STATES

## Abstract

**Background:**

Each year, over 16,000 patients die from malignant brain cancer in the US. Long noncoding RNAs (lncRNAs) have recently been shown to play critical roles in regulating neurogenesis and brain tumor progression. To better understand the role of lncRNAs in brain cancer, we performed a global analysis to identify and characterize all annotated and novel lncRNAs in both grade II and III gliomas as well as grade IV glioblastomas (glioblastoma multiforme [GBM]).

**Methods and Findings:**

We determined the expression of all lncRNAs in over 650 brain cancer and 70 normal brain tissue RNA sequencing datasets from The Cancer Genome Atlas (TCGA) and other publicly available datasets. We identified 611 induced and 677 repressed lncRNAs in glial tumors relative to normal brains. Hundreds of lncRNAs were specifically expressed in each of the three lower grade glioma (LGG) subtypes (IDH1/2 wt, IDH1/2 mut, and IDH1/2 mut 1p19q codeletion) and the four subtypes of GBMs (classical, mesenchymal, neural, and proneural). Overlap between the subtype-specific lncRNAs in GBMs and LGGs demonstrated similarities between mesenchymal GBMs and IDH1/2 wt LGGs, with 2-fold higher overlap than would be expected by random chance. Using a multivariate Cox regression survival model, we identified 584 and 282 lncRNAs that were associated with a poor and good prognosis, respectively, in GBM patients. We developed a survival algorithm for LGGs based on the expression of 64 lncRNAs that was associated with patient prognosis in a test set (hazard ratio [HR] = 2.168, 95% CI = 1.765–2.807, *p* < 0.001) and validation set (HR = 1.921, 95% CI = 1.333–2.767, *p* < 0.001) of patients from TCGA. The main limitations of this study are that further work is needed to investigate the clinical relevance of our findings, and that validation in an independent dataset is needed to determine the robustness of our survival algorithm.

**Conclusions:**

This work identifies a panel of lncRNAs that appear to be prognostic in gliomas and provides a critical resource for future studies examining the role of lncRNAs in brain cancers.

## Abstract 2

In an in silico study, Anindya Dutta and colleagues study the expression of long noncoding RNAs in brain tumors and normal brain tissue and the possible implications for disease prognosis.

## Author Summary

**Why Was This Study Done?:**

- Long noncoding RNAs (lncRNAs) have recently been shown to play a crucial role in normal physiology as well as various disease states; however, the role of lncRNAs in gliomas has not been well characterized.
- This study was undertaken to determine to what extent lncRNAs are dysregulated in glial tumors, whether lncRNA expression can be used to assess patient prognosis, and to determine which of the thousands of newly discovered lncRNAs should be prioritized for mechanistic studies.

**What Did the Researchers Do and Find?:**

- We analyzed over 700 publicly available glioma, glioblastoma, and normal brain RNA sequencing datasets and identified hundreds of lncRNAs with altered expression in gliomas or glioblastomas relative to normal brain tissue.
- The expression of many lncRNAs was found to be associated with a tumor’s mutational status as well as its molecular subtype.
- Using lncRNA expression and Cox regression modeling, we developed a survival algorithm that was able to separate glioma patients into two distinct prognostic groups. Several lncRNAs were identified that also predicted different outcomes in glioblastomas.

**What Do These Findings Mean?:**

- Our analysis provides an important resource for studying lncRNAs in glial tumors by helping prioritize which lncRNAs to investigate for disease relevance.
- An lncRNA panel could potentially be used in the future to help distinguish glioma patients with good versus poor prognosis.

## Introduction

Malignant gliomas are the most common aggressive primary brain tumor, with nearly 23,000 new cases diagnosed each year in the US [1]. The most aggressive malignant gliomas, anaplastic astrocytoma and glioblastoma multiforme (GBM), have 5-y survival rates of 23% and 5%, respectively. World Health Organization grade II and III gliomas are less aggressive than grade IV glioblastomas (GBMs), and have been grouped together by The Cancer Genome Atlas (TCGA) as lower grade gliomas (LGGs). Once thought to be a single disease, GBMs are now recognized as having a considerable level of intertumor heterogeneity, and studies have found that GBMs can be subdivided into four subtypes, proneural, neural, classical, and mesenchymal, based on their transcriptional profile [2,3]. Importantly, these subtypes are associated with differing clinical outcomes, including varying responses to intensive therapy and differences in overall survival [3]. Similar to GBMs, LGGs can be categorized into distinct subtypes, IDH1/2 mut, IDH1/2 mut 1p19q codeletion, and IDH1/2 wt, based on IDH1/2 mutational status and the presence of a codeletion of 1p19q [4]. Each subtype has distinct clinical phenotypes, with the IDH1/2 wt subtype being the most aggressive and dissimilar to the other LGG subtypes [4,5]. Although knowledge of tumor subtype has clinical utility, the best prognostic indicator for patients with glial tumors is the mutational status of IDH1 and IDH2 [6]. In LGGs, patients with wild-type IDH1/2 have a median survival of 1.7 y, while those with mutant IDH1/2 have a median survival between 6.3 and 8.0 y. In GBMs, the corresponding median survival estimates are 1.1 and 2.1 y for wild-type and mutant IDH1/2, respectively [4].

Proteins have been thought to be the primary functional effectors of cells until relatively recently, when the roles of noncoding RNAs (ncRNAs) began to be appreciated for their contributions to most biological processes. Spurred by large sequencing consortia such as ENCODE and FANTOM, interest in ncRNAs has grown rapidly, in part due to the discovery that the vast majority of the mammalian genome is transcribed and that most of the resulting transcripts do not code for proteins [7–9].

Long noncoding RNAs (lncRNAs) are a class of ncRNAs greater than 200 bp in length that do not code for a protein. lncRNAs have mechanistically diverse functions in the cell, and in the nucleus have been shown to regulate gene expression either in cis or in trans by recruiting chromatin-modifying complexes to promoters of target genes [10,11]. Also, lncRNAs have been found to regulate gene expression by promoting long-distance genomic interactions [12]. Other, mainly cytoplasmic, lncRNAs have been shown to regulate the protein concentrations produced from target genes in part by affecting mRNA stability or the translational efficiency of an mRNA [13–15].

Recent work has shown that lncRNAs play a critical role in various biological pathways including the immune system [16], muscle differentiation [17,18], neural lineage commitment, lineage specification, and synaptogenesis [19–23]. In addition to their role in normal physiological processes, lncRNAs are also important regulators of disease processes [24,25]. In cancer, lncRNAs can act as either tumor suppressors or oncogenes, and have been shown to regulate tumor growth and metastasis in breast, prostate, and liver cancer [10,26–28]. Although some lncRNAs have been linked to brain tumor development and pathogenesis, the overall study of lncRNAs in brain tumors has lagged behind [29–32]. In this study, we sought to categorize all dysregulated lncRNAs in glial tumors and to identify lncRNAs that are associated with patient prognosis.

## Methods

### Ethics Statement

All patient samples, including 15 primary GBM specimens and five normal brain specimens, were obtained from consented patients undergoing surgical treatment at the University of Virginia and were acquired in accordance with a protocol approved by the University of Virginia’s institutional review board.

### Planning the Analysis

There was no protocol or prospective analysis plan for the study. Unaligned sequencing reads for TCGA GBMs and LGGs were downloaded from the Cancer Genomics Hub. Normal brain sample SRA files (SRP033725, SRP045638, SRP044668, and SRP048683) [33–36] were downloaded from the Sequence Read Archive (SRA) database on October 4, 2014. Most RNA sequencing (RNA-seq) samples in TCGA originate from patients from the US; however, TCGA collects patient samples from other countries as well, including Canada, Russia, and Italy. The LGG survival algorithm was devised in November 2015; 70% of patients were used as a test set, and 30% were retained for a validation set. Following suggestions by a reviewer in September 2016, this division was subsequently changed to 60% of patients in the test set and the remaining 40% in the validation set.

### Identification of Novel lncRNAs and Quantification of lncRNA Abundance

The aforementioned SRA files were converted to fastq files using the SRA Toolkit v2.3.5. All fastq files were aligned to the hg38 reference genome with Tophat2 using default settings [37]. Novel transcripts (transcripts not found in reference transcript annotation files: GENCODE and RefSeq) were identified in each sample using Cufflinks2 de novo assembly [38]. A consensus transcript assembly was generated using Cuffmerge. Novel transcripts whose genomic coordinates did not intersect with known transcripts from a custom GTF file consisting of transcripts from GENCODE v21, RefSeq, and Cabili et al. [39] were kept for further validation.

We determined the coding potential for each novel transcript using CPAT (Coding-Potential Assessment Tool) and intersection with a mass spectrometry database. First, the in silico coding potential of each novel transcript was assessed using CPAT [40], and any transcripts with a CPAT score above 0.5242 were considered transcripts of unknown coding potential and were not included in downstream analysis. Second, we mapped all unique peptides from the ProteomicsDB [41] mass spectrometry database to all known proteins. All potential ORFs within each novel transcript were translated, and all unmapped peptides were mapped on the translated ORFs. Any novel transcript with more than one mapped peptide was not considered for downstream analysis. All novel transcripts that met these criteria were considered as novel lncRNAs and added to our finalized GTF file. Using the finalized GTF file, the expression of all genes was quantified using Cuffquant and Cuffnorm.

### Validating lncRNA Expression in Clinical Samples

Fresh-frozen GBM and normal brain tissue samples were obtained from the University of Virginia, and RNA was isolated using Trizol (Thermo Fisher). RNA treated with DNase (RQ1 Promega, Thermo Fisher) was used for reverse transcription with SuperScript III (Thermo Fisher). Quantitative real-time PCR (RT-PCR) was performed on tissue cDNA using SYBR Green (Thermo Fisher), and the expression of LINC00152, TUNAR, and LINC01476 was normalized to that of the housekeeping gene encoding actin.

### Identifying Differentially Expressed lncRNAs and lncRNAs Associated with Mutation Status and Subtype

To identify differentially expressed lncRNAs, we selected only lncRNAs with a median expression greater than 0.5 fragments per kilobase of exon per million fragments mapped (FPKM); 4,288 lncRNAs in LGGs and 3,297 lncRNAs in GBMs met this threshold and were used in our downstream analysis. Expression values for each lncRNA in 170 GBM samples, 497 LGG samples (we removed 16 samples from our initial pool of 513 LGGs due to >15% of transcripts having expression values three standard deviations above or below the mean expression for all LGGs), and 78 normal tissue samples were used to calculate the Kolmogorov-Smirnov test (KS test) statistic [42]. lncRNAs with a Benjamini-Hochberg-corrected false discovery rate (FDR) [43] of <0.05 and a fold change greater than or equal to four were considered differentially expressed. The correlation between copy number variation (CNV) and lncRNA dysregulation was determined by calculating the Spearman correlation coefficient between lncRNA expression and the copy number segment mean (Tier 3 TCGA data accessed from the Broad GDAC Firehose; https://gdac.broadinstitute.org) of the genomic region that gives rise to each lncRNA. lncRNAs with Spearman correlation coefficients greater than or equal to 0.2 were considered to be correlated. The overlap between GBM and LGG differentially expressed lncRNAs was measured using the Jaccard index, a statistic that compares the similarity and divergence of two datasets, defined as the intersection of two datasets divided by the union of the datasets:

$$J(A,B)=\frac{A\cap B}{A\cup B}.$$

To identify lncRNAs associated with individual somatic mutations, we separated patients into two groups based on the presence of nonsynonymous mutation or a non-inframe insertion or deletion in commonly mutated protein-coding genes (prevalence of 5% or greater). Differential expression was measured using the same statistical method mentioned above, with a log2 fold change of greater than 0.5 as a cutoff.

Subtype-associated lncRNAs were selected by separating tumors into groups based on their subtype and using the KS test statistic to determine if the expression of a given lncRNA was different in a specific subtype (FDR < 0.05). Specifically, a lncRNA was considered subtype specific only if its expression was statistically different from each other subtype in a paired comparison. GBMs were separated into subtypes based on predesignated subtyping (Cancer Genome Browser). LGGs subtypes were determined by identifying the mutational status of IDH1 or IDH2 from TCGA’s preprocessed mutation calling data and identifying LGGs with 1p19q deletions from TCGA’s preprocessed CNV data. The statistical significance of the overlap between GBM and LGG subtype-specific lncRNAs was determined by comparing the observed lncRNA overlap to the overlap obtained through 1,000 random iterations of the two sample sets, keeping the number of lncRNAs in each sample equal to that in the observed subtype-specific lncRNA sets.

### LGG Survival Prediction

To create a lncRNA survival model for LGGs, we first randomly selected 60% of patients to serve as a test set and reserved the remaining 40% of patients for independent validation. Random subsamples of 66% of the test set of patients were subjected to a multivariate Cox regression [44] survival model. This was repeated with 100 random subsamples from the test set of patients. Age, grade, sex, IDH1/2 mutational status (S7 Table), and inverse normalized lncRNA expression levels were used as variables in the LGG survival model. In total, 64 lncRNAs had statistically significant Cox coefficients in 80% of the 100 subsamples and were included in our survival algorithm. To combine the predictive power of the prognostic lncRNAs, the following steps were taken. For every patient in our test set, the expression of each prognostic lncRNA was compared to the average expression of that lncRNA in patients from the test set. If the absolute value of the expression *Z*-score of a given lncRNA was ≥1, the median Cox coefficient of that lncRNA from the 100 subsamples was added to a summed Cox coefficient, and this was repeated for each of the prognostic lncRNAs (S2 and S3 Figs). Patients were divided into two groups based on whether the summed Cox coefficient was positive (poor prognosis) or negative (good prognosis), consistent with the interpretation that a Cox coefficient > 0 indicates a poor outcome and a Cox coefficient < 0 indicates a good outcome. The survival differences of the groups were displayed on a Kaplan-Meier survival curve. We independently applied this survival algorithm, as mentioned above, to the validation patient set (the retained 40% of patients).

### lncRNAs Associated with Survival in GBM and GBM Subtypes

Prognostic lncRNAs were identified using a Cox proportional hazard model similar to that stated above, except that we included age, sex, and inverse normalized lncRNA expression in the survival model. lncRNAs that were associated with prognosis (*p*-value < 0.05) were then separated based on whether they predicted a poor or good prognosis. We predicted the possible pathways that each lncRNA is involved in using guilt-by-association analysis, as previously described [45]. To identify lncRNAs that predict survival in each subtype, we performed Cox regression for each lncRNA in a given subtype (based on subtypes specified by the Cancer Genome Browser) and selected only lncRNAs with a *p*-value of ≤0.05.

## Results

### Identifying Novel lncRNAs

**Fig 1:**
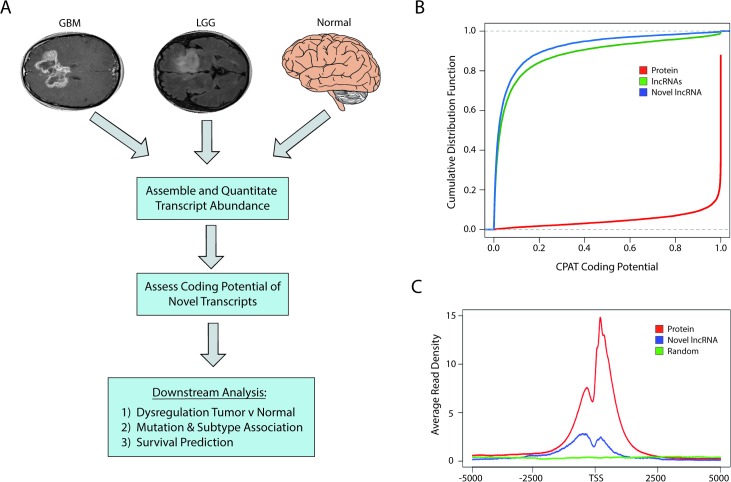
Identification of novel lncRNAs in glial tumors and normal brain RNA-seq samples. (A) Overview of analysis pipeline for identifying novel lncRNAs and determining their associations with clinically relevant phenotypes. (B) Cumulative distribution function plot of CPAT scores demonstrates that the majority of novel transcripts are predicted to not code for proteins (CPAT score < 0.5242). (C) Metagene plot of H3K4me3 ChIP-seq data from U87 cell samples shows enrichment in promoters of protein-coding genes and novel lncRNAs but not in a randomized genomic control. GBM, glioblastoma multiforme; LGG, lower grade glioma; lncRNA, long noncoding RNA; RNA-seq, RNA sequencing.

In order to identify and catalog all novel lncRNAs (unannotated lncRNAs) in brain cancers, we used the Tuxedo Suite [37,38] to align, assemble, and quantify the expression of novel and annotated transcripts from 170 GBMs and 497 LGGs originating from TCGA and 78 normal brain samples from both TCGA and publicly available datasets (Fig 1A; S1 Table). We initially filtered all novel transcripts from the consensus transcriptome that (1) overlapped with any annotated transcript, (2) were less than 200 bp, or (3) did not contain a splice junction. We next assessed the coding potential of all novel transcripts using both in silico predictions as well as intersection with a human proteome database with peptide data from over 100 cell lines and 60 tissues that, importantly, includes brain tissue [41].

CPAT (Coding-Potential Assessment Tool) determines the coding potential of a novel transcript based on relative ORF size, codon bias, and nucleotide hexamer bias. Any transcripts with a CPAT score ≥ 0.5242—a threshold that separates noncoding RNAs and protein-coding genes [46]—were removed from further consideration (Fig 1B). We next sought to determine if there was any biological evidence of protein products derived from the novel transcripts by parsing data from ProteomicsDB [41]. Peptides from the database were first aligned to all known proteins, and any unaligned peptide was then aligned to all translated ORFs within the novel transcripts. Any transcript with two or more mapped peptides, suggesting that it could potentially be a novel protein-coding gene, was removed from downstream analysis. Only seven novel transcripts had more than two aligned peptides and were removed from our downstream analysis, which is in line with other studies reporting low levels of spurious ribosomal associations with ncRNAs [47]. After filtering our list of novel transcripts, we identified over 2,700 novel lncRNAs. After cataloging the novel lncRNAs expressed in LGGs, GBMs, and normal brain, the analyses were carried out with the entire pool of lncRNAs (both novel and annotated) whose average expression was greater than 0.5 FPKM.

Similar to protein-coding genes, lncRNAs are often transcribed by RNA polymerase II and share similar active chromatin marks of H3K4me3 on their promoters. We therefore tested whether H3K4me3 was located at the promoters of the novel lncRNAs identified in this study, using H3K4me3 ChIP-seq data from U87 cells. The promoters of the novel lncRNAs were enriched in H3K4me3 chromatin marks relative to a randomized genomic control, although to a lesser extent than the promoters of protein-coding genes (Fig 1C).

### Dysregulation of lncRNAs in Brain Cancers

Although recent work has begun to address the role of lncRNAs in brain tumors, very few lncRNAs have been found to be dysregulated in glial tumors [30,48]. Therefore, we sought to form a comprehensive list of all lncRNAs whose expression is significantly altered in brain tumors. TCGA RNA-seq data for glial tumors have very few accompanying normal brain samples, making comparisons between normal and tumor groups difficult due to a lack of adequate sample size. To bolster our ability to identify dysregulated lncRNAs in glial tumors, we included RNA-seq data from publicly available normal brain samples that were obtained from regions of the brain where glial tumors commonly arise (e.g., cortex, and excluding regions such as hippocampus and cerebellum; see Methods).

Using our normal brain samples as a comparison, we tested whether any lncRNAs were over- or underexpressed in GBMs or LGGs relative to normal brain. We identified 454 upregulated and 642 downregulated lncRNAs in GBMs and 403 upregulated and 340 downregulated lncRNAs in LGGs that all had FDRs of <0.05, a statistic that takes into account errors that may arise from multiple testing (see Methods), and had fold changes greater than four (S1 Table). Of these dysregulated lncRNAs, over 80 were newly identified in this study. We tested whether the expression differences of the dysregulated lncRNAs could be explained by genomic CNV in GBMs and LGGs. Consistent with previous studies [49], only a fraction (19% and 20% from GBMs and LGGs, respectively) of the differentially expressed lncRNAs were associated (Spearman correlation coefficient of 0.2 or greater) with tumor CNV (S1 Fig). This suggests that other mechanisms, in addition to CNV, play a role in regulating the changes in lncRNA expression observed in glial tumors.

**Fig 2:**
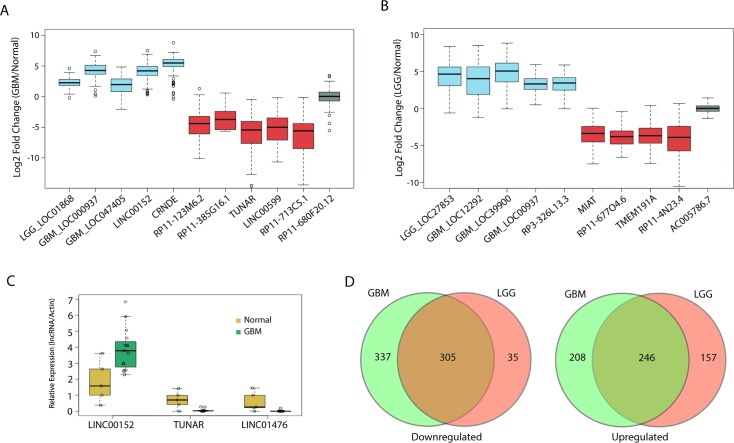
Large alterations in lncRNA expression in glial tumors. (A) Boxplot of ten candidate lncRNAs that are differentially expressed in GBMs compared to normal tissue (blue = upregulated, red = downregulated, grey = no change). (B) Boxplot of nine candidate lncRNAs that are differentially expressed in LGGs compared to normal tissue (blue = upregulated, red = downregulated, grey = no change). (C) Real-time PCR of LINC00152, LINC01476, and TUNAR in 15 GBM and five normal brain samples confirms upregulation of LINC00152 and downregulation of LINC01476 and TUNAR in GBMs compared to normal brain. Expression values for each lncRNA are normalized to that of the gene encoding actin. (D) Large overlap in differentially expressed lncRNAs between GBMs and LGGs. GBM, glioblastoma multiforme; LGG, lower grade glioma; lncRNA, long noncoding RNA.

We have highlighted several differentially expressed lncRNAs in GBMs and LGGs in Fig 2A and 2B, respectively. Of note, our analysis confirmed previous work that identified a lncRNA, CRNDE, that is upregulated in a number of tumors, including GBMs [29]; in our analysis, CRNDE is upregulated over 40-fold in GBMs compared to normal brain (Fig 2A). Furthermore, we also identified TUNAR as being severely downregulated in all glial tumors, almost 45-fold in GBMs and 14-fold in LGGs (Fig 2A and 2B). This is interesting, as other work has shown that TUNAR is a crucial positive regulator of neuronal development and differentiation in zebrafish, mice, and humans, which suggests that brain tumors require the downregulation of TUNAR in order to gain oncogenic properties and escape the restrictions on neuronal cell growth [50,51]. In order to further validate our analysis, we measured by RT-PCR the expression of one lncRNA, LINC00152, which is upregulated 20-fold in GBMs in TCGA data (Fig 2A). Using normal brain and GBM tumor tissue from patients at the University of Virginia, we validated the altered expression of three lncRNAs, LINC00152, TUNAR, and LINC01476. LINC00152 was found to have 3-fold higher expression in tumor tissue relative to normal brain tissue (Fig 2C). TUNAR and LINC01476, which both have lower expression in GBMs relative to normal brain tissue in TCGA, were found to have 12-fold and nearly 100-fold higher expression in normal brain tissue compared to GBM tissue, respectively (Fig 2C).

We next tested whether there is any overlap between the differentially expressed lncRNAs in GBMs and LGGs. Interestingly, there was a large degree of overlap in both the upregulated and downregulated lncRNAs, with a Jaccard index (described in Methods) of 0.4 and 0.45, respectively (Fig 2D). Unlike other tumors, whose tumor grades are more commonly viewed as being along a disease continuum, GBMs and their grade II and III counterparts are not typically regarded as being different stages of a single disease [49]. However, our results suggest that there is a great deal of similarity in the lncRNA profile of GBMs and LGGs. Some of the overlap could be due to the need of glial tumors to downregulate genes related to the differentiation of glia or neurons, though it is unlikely that such de-differentiation would account for such a high degree of overlap between LGGs and GBMs.

### lncRNAs Associated with Patient Tumor Mutation Status

Somatic mutations are well-known drivers of tumorigenesis, and their profound effects on the cell’s transcriptional landscape have been well characterized [52–54]. Although most studies have focused on changes in protein-coding gene expression, recent work has begun to show that somatic mutations can lead to large alterations in lncRNA expression as well [55–57]. Through the TCGA consortium, many recurrent somatic mutations have been identified in GBMs and LGGs, many of which are shared between the two groups [4,58]. In order to determine what effect these mutations might have on the lncRNA transcriptome, for each commonly mutated gene (S7 Table), we separated patients into groups based on their tumor mutational status and then tested whether the expression of any lncRNA is altered in GBMs and LGGs harboring each common somatic mutation.

**Fig 3:**
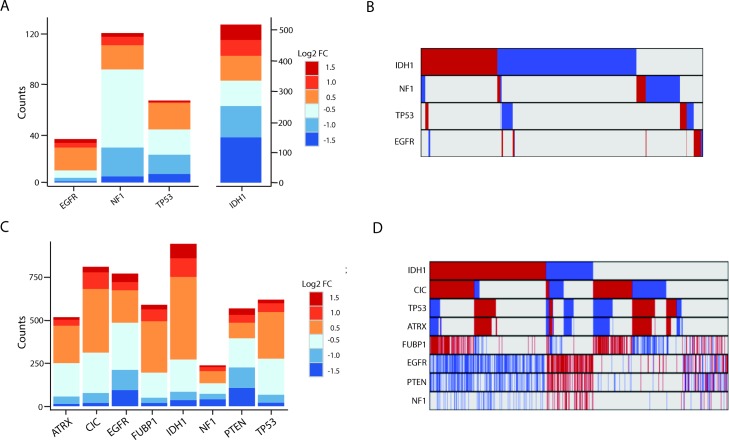
Many lncRNAs are associated with common mutations in GBMs and LGGs. (A) Stacked bar graph of mutation-associated lncRNAs in GBMs. (B) Minimal overlap between mutation-associated lncRNAs in GBMs (red = upregulated, blue = downregulated, grey = no change). (C) Stacked bar graph of mutation-associated lncRNAs in LGGs shows robust deregulation depending on tumor mutational background. (D) Moderate overlap between mutation-associated lncRNA expression trends in GBMs; however, each group of mutation-associated lncRNAs represents a distinct set of GBMs (red = upregulated, blue = downregulated, grey = no change). GBM, glioblastoma multiforme; LGG, lower grade glioma; lncRNA, long noncoding RNA.

We identified hundreds of lncRNAs that were differentially expressed (as described in Methods) in mutated versus non-mutated GBMs and LGGs (Fig 3A and 3C; S2 and S3 Tables). Interestingly, in GBMs there was little overlap in the differentially expressed lncRNAs between different somatic mutation groups (Fig 3B). In contrast, LGGs had a higher degree of overlap between mutation-associated lncRNAs (Fig 3D).

### lncRNAs Associated with Cancer Subtypes

**Fig 4:**
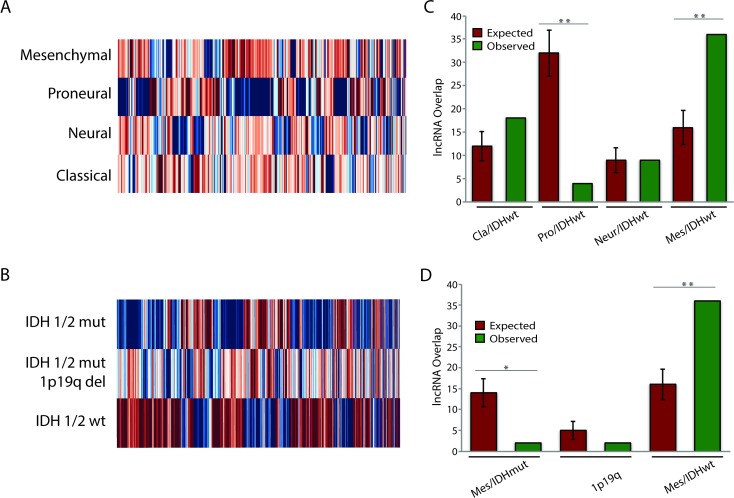
Identification of subtype-associated lncRNAs reveals similarities between GBM and LGG subtypes. (A) Heatmap of all lncRNAs in GBMs with subtype-specific expression patterns demonstrates large expression differences between GBM subtypes. (B) Heatmap of all lncRNAs in LGGs with subtype-specific expression patterns demonstrates large expression differences between LGG subtypes. (C) Overlap of IDH1/2 wt LGG subtype-specific lncRNAs with GBM subtype-specific lncRNAs reveals similarities between mesenchymal GBMs and IDH1/2 wt LGGs. (D) Overlap of GBM mesenchymal subtype-specific lncRNAs with each group of LGG subtype-specific lncRNAs reveals similarities between mesenchymal GBMs and IDH1/2 wt LGGs. * *p* < 0.00005, ** *p* < 0.00001. GBM, glioblastoma multiforme; LGG, lower grade glioma; lncRNA, long noncoding RNA.

Work by TCGA and others has found that both GBMs and LGGs are not homogenous collections of tumors, but can rather be categorized into separate subtypes [2–5]. Each of the glial tumor subtypes is clinically distinct, and understanding the lncRNAs associated with a particular subtype could help to better distinguish between the groups or possibly identify novel therapeutic targets. To this end, we separated patients based on their tumor subtype (S7 Table) and determined whether there were any lncRNAs that were specifically expressed in a given subtype. We identified 64, 211, 95, and 71 lncRNAs that were specifically up- or downregulated in neural, proneural, mesenchymal, and classical GBMs, respectively (Fig 4A; S4 Table). Thirteen of these lncRNAs were novel lncRNAs identified in this study. Furthermore, 1,357, 1,216, and 466 lncRNAs were specifically up- or downregulated in IDH1/2 wt, IDH1/2 mut, and IDH1/2 mut 1p19q codeletion LGGs, respectively (Fig 4B; S5 Table).

Traditionally, GBMs and LGGs have been viewed as distinct oncological entities; however, recent work has begun to suggest that IDH1/2 wt LGGs might be more similar to GBMs than to their IDH1/2 mut LGG counterparts [49]. In order to better understand these similarities, we tested whether there is significant overlap (as described in Methods) between the differentially expressed genes (DEGs) of the IDH1/2 wt LGGs and the DEGs for each GBM subtype. Although there was no statistically significant overlap in DEGs between the neural GBM subtype and the IDH1/2 wt LGG subtype, the proneural GBM subtype had much less overlap with the IDH1/2 wt LGG subtype than would be expected by random chance (Fig 4C). This finding is consistent with the fact that proneural GBMs frequently have point mutations in IDH1/2 [3]. There was a slight increase in the overlap between classical GBM subtype DEGs and IDH1/2 wt LGG DEGs compared to the random model; however, this difference was not statistically significant (*p* = 0.055). Surprisingly, DEGs in mesenchymal GBMs had much higher overlap with DEGs in IDH1/2 wt LGGs compared to the random model (Fig 4C). We next determined whether the overlap between mesenchymal GBMs and IDH1/2 wt LGGs is specific to this LGG subtype or is found with the other LGG subtypes, by measuring the overlap of mesenchymal differentially expressed lncRNAs with differentially expressed lncRNAs from each LGG subtype. In contrast to the greater degree of overlap with the IDH1/2 wt subtype, both the IDH1/2 mut and IDH1/2 mut 1p19q codeletion subtypes had less overlap than would be expected by random chance (Fig 4D). These similarities in the lncRNA profiles of IDH1/2 wt LGGs and mesenchymal GBMs suggest that LGGs with wild-type IDH1/2 may progress to mesenchymal GBMs.

### lncRNA Expression and Survival in LGG Patients

**Fig 5:**
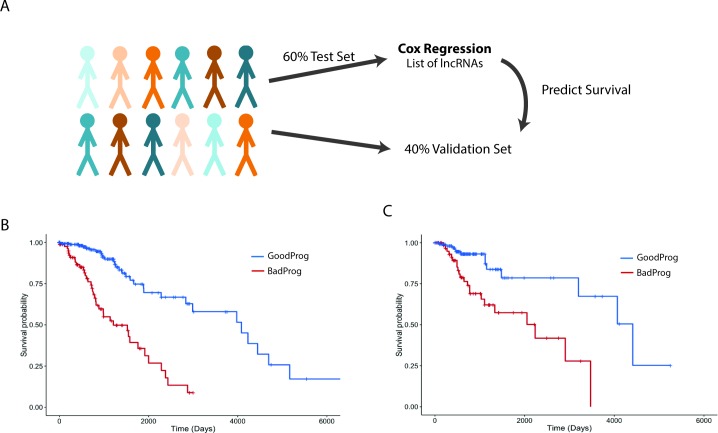
Expression of a subset of lncRNAs is associated with survival in LGG patients. (A) Schematic of survival analysis: 60% of LGG patients were randomly selected to be the test set used to find survival-associated lncRNAs using Cox regression analysis. A summed Cox coefficient derived from 64 survival-associated lncRNAs (selected as in S3 Fig) was used to stratify patients in the test set into two survival groups. This same set of 64 lncRNAs was then used to derive the summed Cox coefficient in the validation set to separate them into two survival groups. (B) Survival plot shows the effective separation of patients from the test set into two distinct survival groups, good prognosis (GoodProg) and poor prognosis (BadProg), based on each patient’s summed Cox coefficient of the 64 lncRNAs (hazard ratio [HR] = 2.168, 95% CI = 1.765–2.807, *p* < 0.001). (C) The summed Cox coefficient for the same 64 lncRNAs is capable of separating patients from the validation set into two groups with very distinct survival probabilities (HR = 1.921, 95% CI = 1.333–2.767, *p* < 0.001). LGG, lower grade glioma; lncRNA, long noncoding RNA.

The main prognostic variable for patients with glial tumors is the mutational status of IDH1 or IDH2. In LGGs, recent work has shown that patients whose tumors also harbor 1p19q codeletions have a slightly better overall survival than patients with IDH1/2 mut tumors without 1p19q codeletions [4]. We decided to test whether the expression of lncRNAs can be used to separate patients into distinct survival groups, independent of IDH1/2 mutational status. To this end, we performed survival analysis utilizing a multivariate Cox proportional hazard model that included IDH1/2 mutational state, age, sex, tumor grade, and lncRNA expression as independent variables in the survival model. It is common to find extreme outliers in large RNA-seq datasets, which can negatively impact survival regression analysis. In order to correct for these outliers, the expression values for each lncRNA were inverse normal transformed, a procedure that increases the sensitivity and specificity of regression analysis using RNA-seq expression values [59]. To attempt to separate patients into distinct prognostic groups using lncRNAs, we randomly assigned 60% of LGG patients with complete clinical data (269 patients) to a test set, on which we performed Cox regression to identify lncRNAs associated with survival in this patient cohort. We then selected all lncRNAs that were significantly associated with survival and created a survival algorithm with variables for each lncRNA that were weighted based on each lncRNA’s contribution to overall survival (see Methods). This survival algorithm was then applied to the remaining 40% of patients (180 patients) who constituted the validation set (Figs 5A, S2 and S3).

After performing Cox regression on our test set, we identified 64 lncRNAs that were consistently associated with survival. These lncRNAs were included in our survival algorithm, which was then applied to the test set. Each patient received a score, based on how many prognostic lncRNAs met our expression cutoff (see Methods), and patients were then divided into two groups, good prognosis and poor prognosis. Our algorithm separated the test set into groups of 85 and 184 patients with median survival times of 1,209 and 4,084 d, respectively (HR = 2.168, 95% CI = 1.765–2.807, *p* < 0.001) (Fig 5B; S6 and S8 Tables). We next applied this survival algorithm to the validation set and were successfully able to separate patients into distinct groups of 66 and 114 patients with median survival times of 2,235 and 4,412 d, respectively (HR = 1.921, 95% CI = 1.333–2.767, *p* < 0.001) (Fig 5C; S6 and S8 Tables).

### Identifying lncRNAs Associated with Survival in GBMs

**Fig 6:**
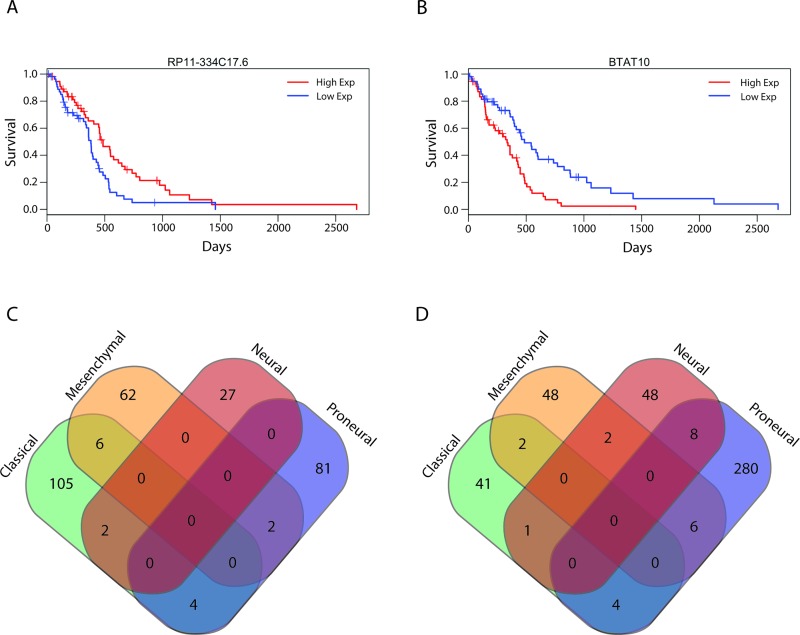
Survival-associated lncRNAs in GBMs and GBM subtypes. (A and B) Representative survival plots of lncRNAs that predict survival in GBMs: RP11-334C17.6 (HR = 0.728, 95% CI = 0.6011–0.883, *p =* 0.00122) (A) and BTAT10 (HR = 1.298, 95% CI = 1.0881–1.548, *p =* 0.00374) (B). (C and D) lncRNAs associated with a poor prognosis (C) and good prognosis (D) in individual subtypes show minimal overlap between subtypes. GBM, glioblastoma multiforme; HR, hazard ratio; lncRNA, long noncoding RNA.

We next sought to identify all lncRNAs that were associated with overall survival in patients with GBMs. Using Cox regression we identified 584 lncRNAs that were associated with a poor prognosis and 282 lncRNAs that were associated with better survival outcomes (S9 Table). A subset of these lncRNAs were independently used to separate GBM patients based on lncRNA expression levels in the top third and bottom third of patients (55 patients), and Kaplan-Meier plots show that these groups were associated with prognosis with statistical significance (Fig 6A and 6B). Patients with high expression of RP11-334C17.6 had a median survival time of 485 d, while patients with low expression had a median survival time of 380 d (HR = 0.728, 95% CI = 0.6011–0.883, *p =* 0.00122) (Fig 6A). Patients with high and low expression of BTAT10 had median survival times of 335 and 485 d, respectively (HR = 1.298, 95% CI = 1.0881–1.548, *p =* 0.00374) (Fig 6B). However, unlike in the LGGs, we have not yet succeeded in combining the individually predictive lncRNAs into a survival algorithm that can predict prognosis in GBMs with statistical significance.

Unlike for proteins, ascertaining the function of a lncRNA based on sequence composition is extremely difficult. However, studies have shown that it is possible to infer what biological pathways a lncRNA might function in using guilt-by-association analysis, a technique that infers association of a lncRNA with a biological pathway based on the pathway enrichment of protein-coding genes whose expression is highly correlated with the lncRNA [45]. We used guilt-by-association analysis to determine what biological pathways are enriched in our positive and negative prognostic lncRNA groups. Interestingly, lncRNAs that are associated with a better prognosis in GBMs are more likely to be associated with signaling pathways, showing enrichment in protein kinase and phosphorylation pathways as well as signal transduction pathways. Conversely, lncRNAs that are associated with poor patient outcomes are highly associated with cell cycle pathways, immune response, and chromosome organization (Table 1).

**Table 1:** Enriched biological pathways associated with prognostic lncRNAs by guilt-by-association analysis.

Good Prognosis Group		Poor Prognosis Group	
Pathways	Number of lncRNAs	Pathways	Number of lncRNAs
Intracellular signaling cascade	67	Response to external stimulus	59
Protein kinase cascade	61	Chromosome organization and biogenesis	55
Response to external stimulus	60	RNA processing	54
Organ development	58	Cell cycle (GO:0007049)	53
G protein coupled receptor protein signaling pathway	56	Cell cycle process	53
Cell surface receptor linked signal transduction	56	Cell cycle phase	52
Defense response	56	Defense response	52
Signal transduction	56	Mitotic cell cycle	51
Protein amino acid phosphorylation	55	Immune response	51
Regulation of signal transduction	55	RNA metabolic process	50

lncRNA, long noncoding RNA.

We next subdivided all of the GBM tumors into their respective subtypes and performed Cox regression with all lncRNAs in each subtype: 165, 128, 88, and 385 lncRNAs were associated with prognosis in classical, mesenchymal, neural, and proneural GBM subtypes, respectively. Given the transcriptional, clinical, and phenotypic differences between the subtypes, we then tested if there was any overlap in the identities of the positive and negative prognostic lncRNAs between subtypes. There was very little overlap noted between the prognostic lncRNAs in each subtype (Fig 6C and 6D), consistent with the hypothesis that the GBM subtypes arise from distinct mutational backgrounds and have very different biology.

## Discussion

Our analysis of RNA-seq data for grade II, III, and IV brain tumors and normal brain tissue has identified hundreds of dysregulated lncRNAs in glial tumors, many of which are associated with tumor subtype or mutational status. Using Cox regression, we identified a panel of 64 lncRNAs that are associated with survival in LGG patients. We also identified lncRNAs that are similarly associated with prognosis in each GBM subtype and found remarkably little overlap of prognostic lncRNAs between GBM subtypes.

The growing appreciation for the important roles that lncRNAs play in tumor development and progression necessitates having a means of prioritizing which lncRNAs should be studied in a given cancer type. Global analyses have been performed for tumor types other than GBMs and LGGs, such as squamous cell lung carcinomas and adenocarcinomas, as well as meta-analyses of all tumors within TCGA [46,57,60]. Although meta-analyses of lncRNAs are extremely important, they have not been especially informative for brain tumors for several reasons. First, due to the broad nature of the analyses, it is not possible to focus on the specific nuances of each tumor type (i.e., subtypes). Second, due to the limited number of normal brain samples in TCGA, GBMs and LGGs were not included in many of the meta-analyses, which relied on comparisons with a reference panel of normal tissues. Lastly, the effect of lncRNA expression on survival in individual tumor types was not a main focus of the studies [46,60]. This is important because, depending on the tumor context, a given lncRNA may act as a tumor suppressor or oncogene [61,62]. By focusing specifically on brain tumors and including over 70 normal brain tissue samples, our analysis provides unique insights into the roles of lncRNAs in aggressive brain cancers.

In addition to studying the roles of annotated lncRNAs, we identified 2,706 novel multi-exon lncRNAs that are present in either normal brain tissue or brain tumors, but are not annotated in the commonly used lncRNA databases (GENCODE and RefSeq). Many of these novel lncRNAs were differentially expressed in brain tumors and were associated with clinically relevant mutations. Although the exact mechanisms leading to altered lncRNA expression are not known, roughly 20% of differentially expressed lncRNAs were weakly correlated with chromosomal amplifications and deletions. We also identified several hundred lncRNAs that were associated with GBM and LGG subtypes. It is well known that IDH1/2 wt LGGs have clinical phenotypes and genomic alterations similar to those of primary GBMs [5]. Interestingly, the intersection of subtype-associated lncRNAs between GBMs and LGGs revealed transcriptional similarities between IDH1/2 wt LGGs and mesenchymal GBMs. Although our analysis suggests an evolutionary link between mesenchymal GBMs and IDH1/2 wt LGGs, it does not preclude other tumor evolutionary pathways leading to the formation of mesenchymal GBMs. Other groups have found evidence that suggests non-GCIMP (non-glioma-CpG island methylator phenotype) mesenchymal GBMs evolve from a proneural GBM precursor [63]. However, this evolutionary pathway does not explain the origin of all mesenchymal GBMs. Our analysis suggests that some mesenchymal GBMs might arise from undetected IDH1/2 wt LGGs, which at clinical presentation would appear to be mesenchymal GBMs.

There are several limitations to this analysis. One limitation is that, because RNA-seq data from TCGA were derived from bulk tumor specimens, we are unable to decipher whether the differences in expression that we have identified are a reflection of alterations in tumor cells’ transcriptional programs or a reflection of tumor heterogeneity and changes in the stromal composition of each individual tumor. Furthermore, although we validated the expression changes of some lncRNAs using independent patient-derived samples, more work is needed to confirm the expression differences we identified for lncRNAs between gliomas and normal brain, and among tumor subtypes and tumors of different mutational status. Another limitation is that the generation and testing of our survival algorithm were performed on the same dataset. Although the “validation” dataset was blinded in the algorithm generation, further validation of our survival analysis in a truly independent dataset is needed to determine the significance and robustness of the survival algorithm. Independent validation of lncRNAs that are associated with survival in GBMs and GBM subtypes is also needed.

As stated earlier, IDH1/2 mutational status is the primary prognostic indicator for glial tumors. Using multivariate survival analysis, we have shown that a panel of lncRNAs can be used to separate LGG patients into distinct prognostic groups. This group of lncRNAs could potentially be used to help identify at-risk patients who might require more intensive therapies, although further validation in an independent dataset is needed to fully test the utility of the survival algorithm. Furthermore, we have also found several hundred lncRNAs that are prognostic in GBMs as a whole, as well as in individual subtypes. In summary, we have performed the first global analysis, to our knowledge, of lncRNAs in LGGs and GBMs. Our analysis can serve as a valuable resource for those working in the field to prioritize which lncRNAs to study in brain cancers.

## Supporting Information

S1 Fig Association of dysregulated lncRNA expression and tumor copy number variation. (A) Histogram of Spearman correlation coefficients for lncRNAs and CNV in GBMs. (B) Histogram of Spearman correlation coefficients for lncRNAs and CNV in LGGs. Red lines indicate Spearman correlation coefficient greater than or equal to 0.2. Blue lines indicate non-correlated lncRNAs.
(TIFF)

S2 Fig Schematic of patient separation for survival algorithm development and validation. (TIFF)

S3 Fig Schematic for creating survival algorithm using lncRNA expression and Cox regression. (TIFF)

S1 Table Median expression and false discovery rates of differentially expressed lncRNAs in GBMs and LGGs. (XLSX)

S2 Table Median expression and false discovery rates of mutation-associated lncRNAs in GBMs. (XLSX)

S3 Table Median expression and false discovery rates of mutation-associated lncRNAs in LGGs. (XLSX)

S4 Table Median expression and false discovery rates of subtype-associated lncRNAs in GBMs. (XLSX)

S5 Table Median expression and false discovery rates of subtype-associated lncRNAs in LGGs. (XLSX)

S6 Table Number of patients at risk in separate Kaplan-Meier plot time intervals for patients belonging to the positive and negative prognostic groups in the test and validation sets. (XLSX)

S7 Table LGG and GBM patient characteristics. (TIFF)

S8 Table Patient characteristics of the test and validation sets of LGG patients. (TIFF)

S9 Table Cox coefficients and *p*-values of prognosis-associated lncRNAs in GBMs. (XLSX)

S1 Text STROBE checklist. (DOC)

## Abbreviations

CNV: copy number variation
DEG: differentially expressed gene
FDR: false discovery rate
FPKM: fragments per kilobase of exon per million fragments mapped
GBM: glioblastoma multiforme
HR: hazard ratio
LGG: lower grade glioma
lncRNA: long noncoding RNA
ncRNA: noncoding RNA
RNA-seq: RNA sequencing
RT-PCR: real-time PCR
TCGA: The Cancer Genome Atlas
