# Supplementary figures and images for: Expression of lncRNAs in Low-Grade Gliomas and Glioblastoma Multiforme: An In Silico Analysis
Source: PLoS Med. 2016 Dec 6;13(12):e1002192. doi: 10.1371/journal.pmed.1002192 (PMC5140055; doi:10.1371/journal.pmed.1002192)

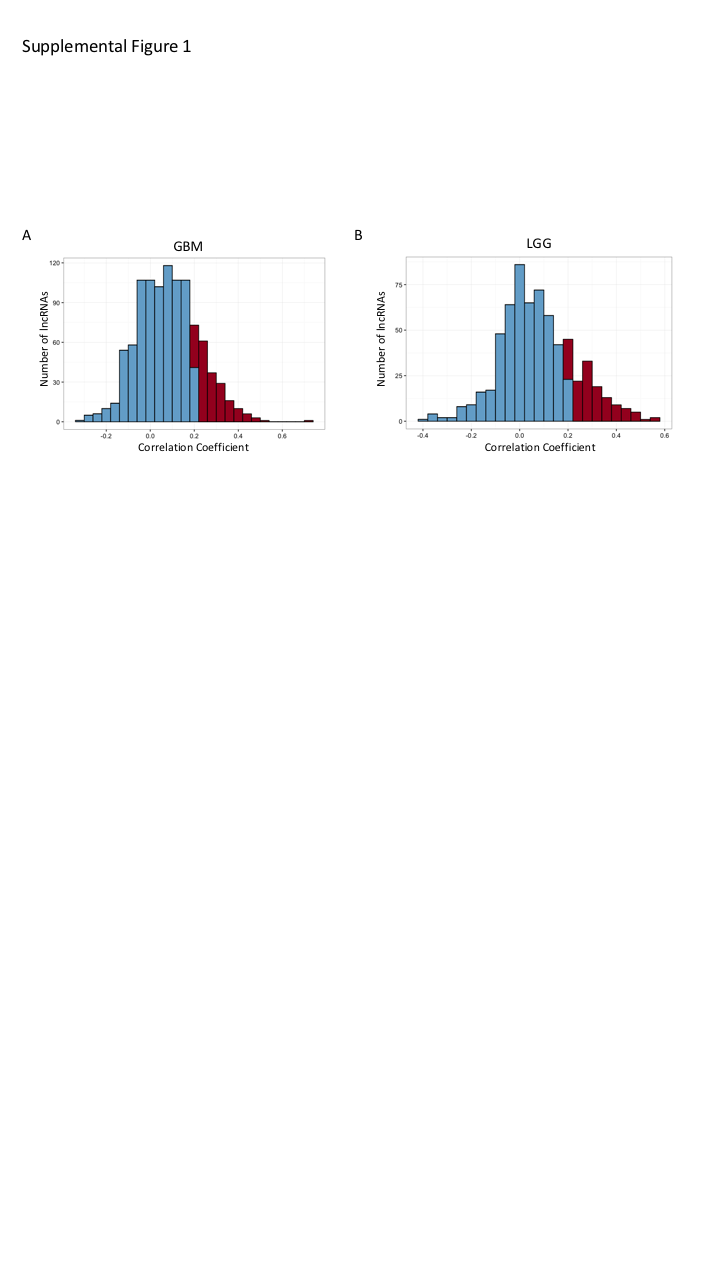

Supplement: S1 Fig — (A) Histogram of Spearman correlation coefficients for lncRNAs and CNV in GBMs. (B) Histogram of Spearman correlation coefficients for lncRNAs and CNV in LGGs. Red lines indicate Spearman correlation coefficient greater than or equal to 0.2. Blue lines indicate non-correlated lncRNAs. (TIFF) [file pmed.1002192.s001.tiff]

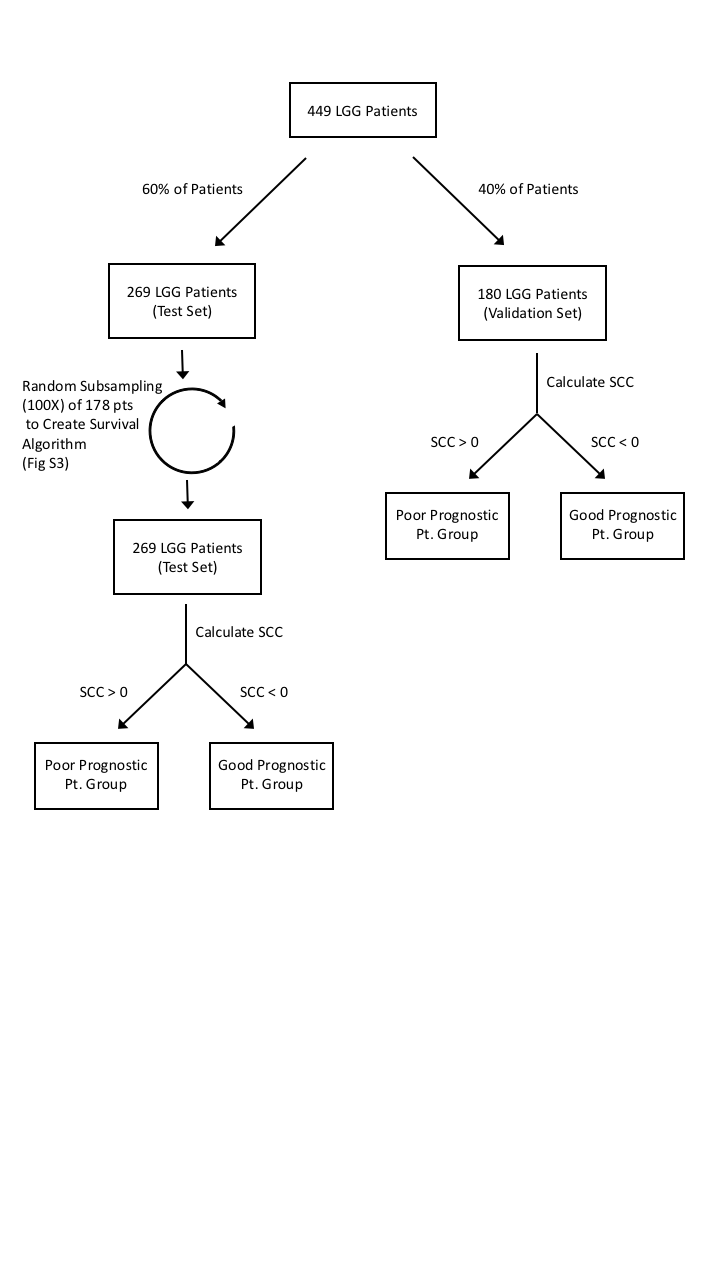

Supplement: S2 Fig — (TIFF) [file pmed.1002192.s002.tiff]

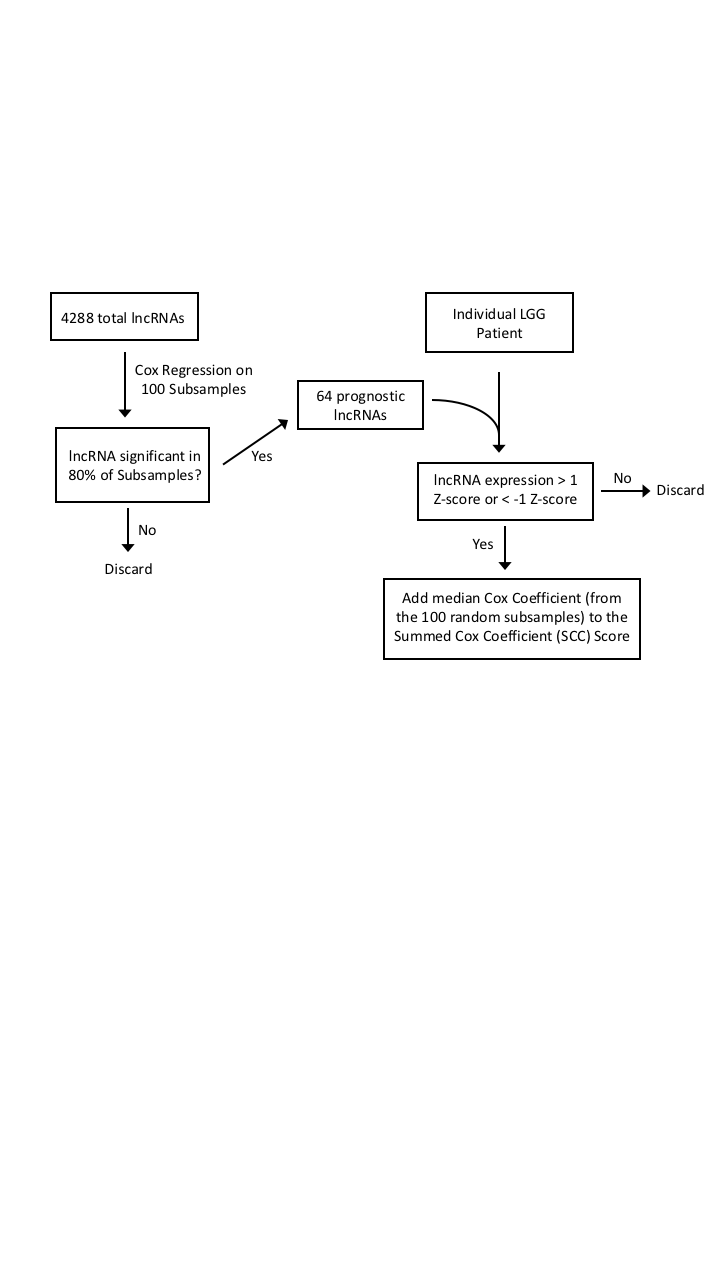

Supplement: S3 Fig — (TIFF) [file pmed.1002192.s003.tiff]

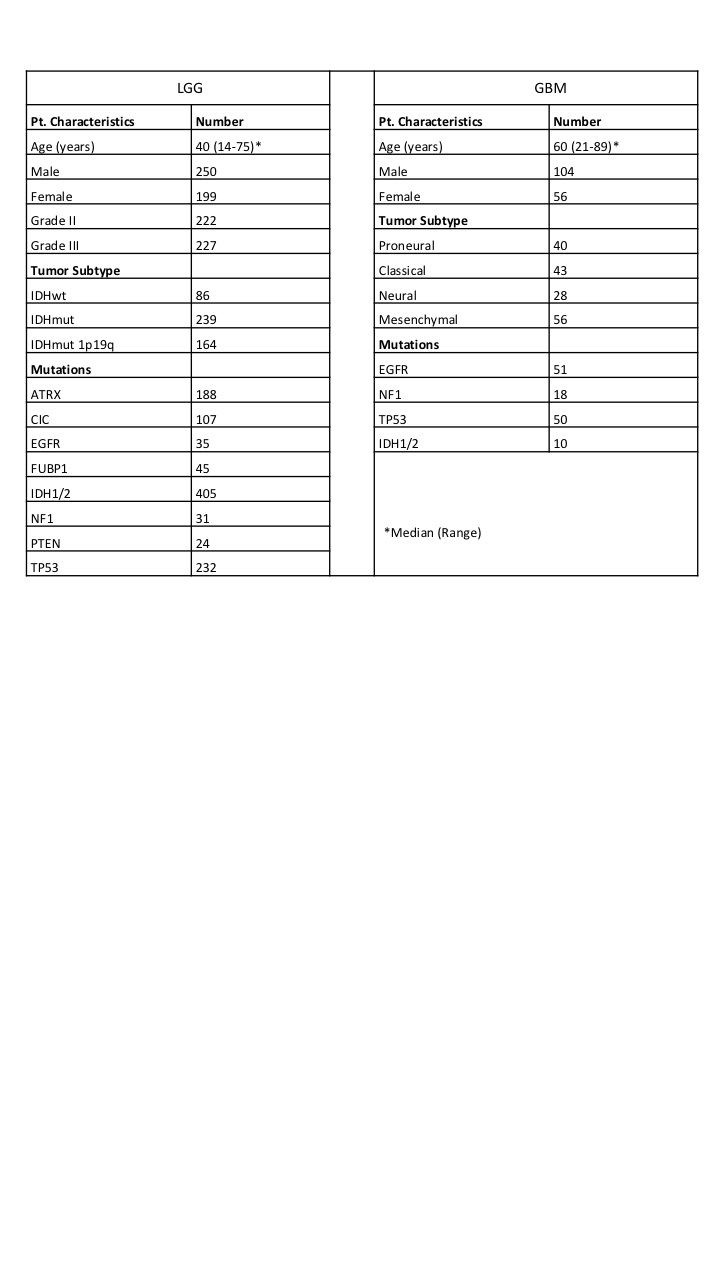

Supplement: S7 Table — (TIFF) [file pmed.1002192.s010.tiff]

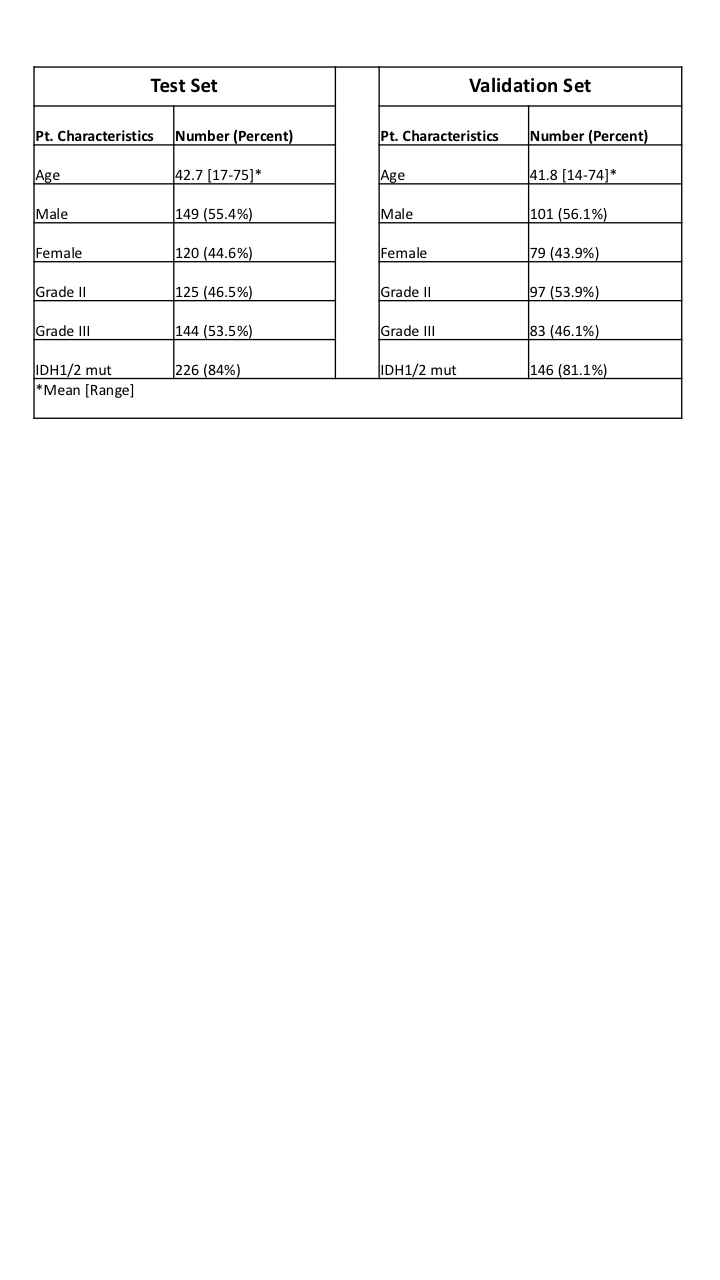

Supplement: S8 Table — (TIFF) [file pmed.1002192.s011.tiff]
