# Supplementary material for: Expression of lncRNAs in Low-Grade Gliomas and Glioblastoma Multiforme: An In Silico Analysis
Source: PLoS Med. 2016 Dec 6;13(12):e1002192. doi: 10.1371/journal.pmed.1002192 (PMC5140055; doi:10.1371/journal.pmed.1002192)
Supplement: S1 Text — (DOC) [file pmed.1002192.s013.doc]

STROBE Statement—Checklist of items that should be included in reports of ***cohort studies***

|  | Item No | Recommendation |
| --- | --- | --- |
| **Title and abstract** | 1 | (*a*) Indicate the study’s design with a commonly used term in the title or the abstract  Title page and Materials and methods section. |
|  |  | (*b*) Provide in the abstract an informative and balanced summary of what was done and what was found  Described in the Abstract |
| Introduction | | |
| Background/rationale | 2 | Explain the scientific background and rationale for the investigation being reported  Described in the Introduction. |
| Objectives | 3 | State specific objectives, including any prespecified hypotheses  Described in the Abstract and at the end of the Introduction. |
| Methods | | |
| Study design | 4 | Present key elements of study design early in the paper  Described in the Results and Materials and Methods section. |
| Setting | 5 | Describe the setting, locations, and relevant dates, including periods of recruitment, exposure, follow-up, and data collection  Described access to publicly available data in the Materials and Methods section. |
| Participants | 6 | (*a*) Give the eligibility criteria, and the sources and methods of selection of participants. Describe methods of follow-up  All data was publicly available as mentioned in the Introduction and Materials and Methods section. |
|  |  | (*b*) For matched studies, give matching criteria and number of exposed and unexposed |
| Variables | 7 | Clearly define all outcomes, exposures, predictors, potential confounders, and effect modifiers. Give diagnostic criteria, if applicable  Mentioned in the Results. |
| Data sources/ measurement | 8* | For each variable of interest, give sources of data and details of methods of assessment (measurement). Describe comparability of assessment methods if there is more than one group  Described in Results and Materials and Methods. |
| Bias | 9 | Describe any efforts to address potential sources of bias  Mentioned in the results and Materials and Methods. |
| Study size | 10 | Explain how the study size was arrived at  We identified the maximum number of applicable publically available RNA-seq data sets. |
| Quantitative variables | 11 | Explain how quantitative variables were handled in the analyses. If applicable, describe which groupings were chosen and why  Described in the Results and in the Materials and Methods. |
| Statistical methods | 12 | (*a*) Describe all statistical methods, including those used to control for confounding  Described in the Results and the Materials and Methods. |
|  |  | (*b*) Describe any methods used to examine subgroups and interactions  Described in the Results and the Materials and Methods. |
|  |  | (*c*) Explain how missing data were addressed |
|  |  | (*d*) If applicable, explain how loss to follow-up was addressed |
|  |  | (*e*) Describe any sensitivity analyses |
| Results | | |
| Participants | 13* | (a) Report numbers of individuals at each stage of study—eg numbers potentially eligible, examined for eligibility, confirmed eligible, included in the study, completing follow-up, and analysed  Described in Results and Materials and Methods, as well as Supplemental Figure 2. |
|  |  | (b) Give reasons for non-participation at each stage |
|  |  | (c) Consider use of a flow diagram  Supplemental Figure 2 |
| Descriptive data | 14* | (a) Give characteristics of study participants (eg demographic, clinical, social) and information on exposures and potential confounders  Patient characteristics are in Supplemental Table 7 and 8 |
|  |  | (b) Indicate number of participants with missing data for each variable of interest  Only used patient with complete clinical data. |
|  |  | (c) Summarise follow-up time (eg, average and total amount) |
| Outcome data | 15* | Report numbers of outcome events or summary measures over time |
| Main results | 16 | (*a*) Give unadjusted estimates and, if applicable, confounder-adjusted estimates and their precision (eg, 95% confidence interval). Make clear which confounders were adjusted for and why they were included  Described throughout the Results section |
|  |  | (*b*) Report category boundaries when continuous variables were categorized |
|  |  | (*c*) If relevant, consider translating estimates of relative risk into absolute risk for a meaningful time period |
| Other analyses | 17 | Report other analyses done—eg analyses of subgroups and interactions, and sensitivity analyses  Described throughout the Results section. |
| Discussion | | |
| Key results | 18 | Summarise key results with reference to study objectives  Described throughout the Discussion. |
| Limitations | 19 | Discuss limitations of the study, taking into account sources of potential bias or imprecision. Discuss both direction and magnitude of any potential bias  Mentioned in the Abstract and the Results section. |
| Interpretation | 20 | Give a cautious overall interpretation of results considering objectives, limitations, multiplicity of analyses, results from similar studies, and other relevant evidence  Presented throughout the results and in the last paragraph of the discussion. |
| Generalisability | 21 | Discuss the generalisability (external validity) of the study results  We have commented on this in the last paragraph of the discussion. |
| Other information | | |
| Funding | 22 | Give the source of funding and the role of the funders for the present study and, if applicable, for the original study on which the present article is based  Listed in the manuscript. |
